# Impact of global budget combined with pay-for-performance on the quality of care in county hospitals: a difference-in-differences study design with a propaensity-score-matched control group using data from Guizhou province, China

**DOI:** 10.1186/s12913-021-07338-8

**Published:** 2021-12-02

**Authors:** Wuping Zhou, Weiyan Jian, Zhifan Wang, Jay Pan, Min Hu, Winnie Yip

**Affiliations:** 1 Department of Health Policy and Management, School of Public Health, Peking University Health Science Center, Beijing, China; 2 West China Research Center for Rural Health Development, West China School of Public Health and West China Fourth Hospital, Sichuan University, Chengdu, China; 3 School of Public Health, Fudan University, Shanghai, China; 4 Harvard School of Public Health, Boston, MA USA

**Keywords:** Global budget, Process quality, County hospitals, Inpatient, China

## Abstract

**Background:**

Provider payment system has a profound impact on health system performance. In 2016, a number of counties in rural Guizhou, China, implemented global budget (GB) for county hospitals with quality control measures. The aim of this study is to measure the impact of GB combined with pay-for-performance on the quality of care of inpatients in county-level hospitals in China.

**Methods:**

Inpatient cases of four diseases, including pneumonia, chronic asthma, acute myocardial infarction and stroke, from 16 county-level hospitals in Guizhou province that implemented GB in 2016 were selected as the intervention group, and similar inpatient cases from 10 county-level hospitals that still implemented fee-for-services were used as the control group. Propensity matching score (PSM) was used for data matching to control for age factors, and difference-in-differences (DID) models were constructed using the matched samples to perform regression analysis on quality of care for the four diseases.

**Results:**

After the implementation of GB, rate of sputum culture in patients with pneumonia, rate of aspirin at discharge, rate of discharge with β-blocker and rate of smoking cessation advice in patients with acute myocardial infarction increased. Rate of oxygenation index assessment in patient with chronic asthma decreased 20.3%. There are no significant changes in other indicators of process quality.

**Conclusions:**

The inclusion of pay-for-performance in the global budget payment system will help to reduce the quality risks associated with the reform of the payment system and improve the quality of care. Future reform should also consider the inclusion of the pay-for-performance mechanism.

**Supplementary Information:**

The online version contains supplementary material available at 10.1186/s12913-021-07338-8.

## Background

Provider payment system has a profound impact on health system performance. Long-term implementation of fee-for-services (FFS) for hospitalization services in rural areas of China, coupled with the “fragmentation” of China’s health system [1], has led to a chronic inefficiency of China’s rural health system with high cost growth, which directly threatens the security of health insurance funds.

In 2016, a number of counties in rural Guizhou, China, implemented the provider payment reform, changing from FFS to global budget (GB), in the hope of reducing the growth rate of healthcare costs. At the beginning of every year, the county hospitals’ total annual budgets were set based on the types of diseases they can treat, the number of hospitalizations and the average cost for each type of disease in the previous year.

At the same time, the design of pay-for-performance is nested, 30% of the total budget is set aside as a bonus for performance assessment. The relative size of the performance payment was based on practice in other parts of China and other country, and this ratio had been shown to produce good results [2–4].

At the middle and end of each year, the competent authority would organize clinical experts to assess the quality of care in the county hospitals. The assessment included the standardization of the treatment process, which were based on international treatment guidelines, and the medical outcomes (for example, the rate of aspirin use in stroke patients, inpatient mortality rate, etc.). The same indicators of the previous year were used as a baseline, and if the quality of care was worse than the previous year, points were deducted. Based on the final score, 30% of the total budget was awarded on a proportional basis.

The effects of the change from FFS to GB, such as a decrease in costs and hospital days, have been more clearly elaborated in many previous studies [5–7]. However, the impact of GB on the quality of care is mixed. In terms of readmission rates, studies based in Maryland have shown that GB led to lower or unchanged readmission rates [8, 9], but the 30-day readmission rate for patients increased 11.4% after the implementation of GB in a Chinese county [10], while Taiwan showed a curious phenomenon that 3-day readmission declined and 14-day readmission rates increased in patients with pneumonia [11]. Song’s long-term observations of Maryland found GB reform to be effective in improving process quality and outcome quality [12–15]. Kan observed cardiovascular patients and found that increasing the intensity of care did not reduce the risk of death, indicating a relative decline in the quality of care [16]. In addition, some studies have reported the satisfaction of patients declined [17, 18].

Other types of provider payment reforms within China such as diagnosis related groups (DRG) have been reported to bring quality risks [19], which arise from the change from FFS to DRG. The change gives providers a tendency to compress care, and when necessary treatments are compressed, quality may be reduced. The GB has a stronger incentive to bring cost control to providers than the DRG [20] and it is more likely to bring quality risk.

In contrast, the strategy of implementing PFP has been shown in many studies to improve quality, an effect that has been observed not only in developed regions [21–23], but also in many developing countries. For example, studies in Afghanistan have shown that pay-for-performance provides incentives for providers to deliver innovative services [24]. Research in Bangladesh showed that pay-for-performance improved the quality of maternal health services [25]. Studies in the Philippines have shown that pay-for-performance improves two types of child health outcomes [26]. Studies in Kenya and Tanzania also confirm that pay for performance can improve the quality of health services [27, 28]. We therefore wanted to know what impact GB combined with PFP would have on the quality of care, and whether PFP can offset the negative impact of GB on quality of care.

## Method

### Study design

Prior to implementing the intervention, 16 representative hospitals were selected by the local government to implement GB, a process that was not completely randomized due to hospital size and administrative planning considerations. Meanwhile, hospitals of similar size and capacity were selected as a control group, from which we obtained deidentified data for 10 hospitals, and we were unable to obtain data from more hospitals due to data security.

In order to clearly define the quality of care, based on previous studies, we have identified four diseases for which there are clear guidelines for treatment and necessary medical services (including drug use and necessary tests), which were: acute myocardial infarction, stroke, pneumonia, and chronic asthma. Also, these four diseases are common critical illness in county hospitals in China [29, 30].

This study used chart review to collect information on the quality of care. Patients with these four diseases were screened by ICD-10 from discharged patients. Cases were judged logically to ensure the quality of the data. Patients aged less than 18 years with stroke, acute myocardial infarction and chronic asthma were not included in this study. This study did not set exclusion criteria for pneumonia.

All data were obtained from patients admitted to the 26 county-level hospitals in Guizhou Province in 2015 and 2016, of which 16 hospitals implemented GB in 2016 and the other 10 had been implementing FFS payment from 2015 to 2016.

### Variables

The quality indicators used in the study refers to the clinical practice guidelines for diseases. Pneumonia quality indicators refer to the 2016 Chinese Medical Association Guidelines for the Diagnosis and Treatment of Pneumonia, which were jointly completed with the advice of experts from the United States and Europe [31]. Quality indicators of acute myocardial infarction refers to the 2010 guidelines of the Chinese Medical Association (CMA), which is consistent with the guidelines of the European Society of Cardiology (2007) and the American College of Cardiology. Quality indicators of chronic asthma refers to the Global Initiative for Asthma 2019 edition, which details the standardized treatment process of chronic asthma in adults [32]. Stroke quality indicators refer to the 2018 edition of the Chinese guidelines for the treatment of acute ischemic stroke, and the revised guidelines refer to the development method of World Stroke Organization guideline and our national conditions and operability [33]. From these guidelines, we selected the services needed to be provided as process quality indicators, and these indicators have been widely used in previous studies in China and abroad, so it is reasonable to use these indicators as process quality indicators.

The quality indicators include:

For pneumonia -- oxygenation index assessment, rate of sputum culture, antibiotic use, whether antibiotics were administered within 6 h of admission, influenza vaccination, pneumococcal vaccination, and smoking cessation advice.

For acute myocardial infarction -- aspirin within 24 h, aspirin at discharge, β-blocker at discharge, smoking cessation advice.

For chronic asthma -- oxygenation index assessment, influenza vaccine, pneumococcal vaccine, smoking cessation advice.

For stroke -- aspirin within 24 h, aspirin at discharge, statin at discharge, smoking cessation advice.

The denominator of “incidence of smoking cessation advice” is those patients who have a smoking history.

### Statistical analysis

First, we conducted a propensity score matched by disease type and age to obtain a control sample on a 1:1 basis (results showed in appendix). The total impact on quality will then be measured by a difference-in-differences method (DID) design. After matching, the sample size was 5118 in the intervention and control groups, with 1297 in each group before intervention and 1262 in each group after intervention.

Then the DID model was used to assess whether payment system reforms affected quality of care for those four diseases. In order to make the model credible, we compared the basic characteristics of patients and the quality of care between the control and intervention groups (results showed in Table 1 and Table 2). The DID model used in the study was as follows.

**Table 1:** Characteristics of patients with different diseases

Variables	pneumonia		AMI		chronic asthma		stoke	
	2015	2016	2015	2016	2015	2016	2015	2016
N	1498	1240	198	254	328	442	570	588
Age, years								
< 19	66.4%	64.4%	0.0%	0.0%	0.0%	0.0%	0.0%	0.0%
19–40	3.7%	3.6%	3.0%	3.2%	0.6%	1.4%	4.9%	0.7%
41–60	9.6%	11.1%	36.4%	31.5%	21.3%	19.5%	41.8%	22.1%
61–80	16.8%	17.4%	41.4%	57.5%	63.4%	62.4%	46.8%	64.3%
> 80	3.5%	3.6%	19.2%	7.9%	14.6%	16.7%	6.5%	12.9%
History of smoke								
Not smoker	97.7%	96.1%	75.8%	83.9%	81.1%	81.2%	83.7%	83.7%
Smoker	2.3%	4.0%	24.2%	16.1%	18.9%	18.8%	16.3%	16.3%

**Table 2:** Process quality indicators for control and intervention groups in 2015 and 2016

Indicators	2015				2016				*DD* *%*	*Pvalue*
	Treatment%	Control %	*Diff.* *%*	*Pvalue*	Treatment%	Control %	*Diff.* *%*	*Pvalue*		
**Pneumonia**										
Oxygenation index assessment	2.54	3.60	−1.06	0.231	3.55	6.94	−3.39	0.007	−2.33	0.005
Rate of sputum culture	13.62	13.75	−0.13	0.940	20.16	11.94	8.22	0.000	8.35	0.007
Antibiotic use	95.19	97.20	−2.01	0.043	96.29	97.26	−0.97	0.335	1.04	0.030
Antibiotic use within 6 h	89.72	94.26	−4.54	0.001	89.68	95.65	−5.97	0.000	−1.43	0.000
Influenza vaccine	0.53	0.40	0.13	0.705	0.00	0.48	−0.48	0.083	− 0.61	0.526
Pneumonia vaccine	0.00	0.00	0.00	0.000	0.00	0.00	0.00	0.000	0.00	0.157
Smoking cessation advice	28.57	23.08	5.49	0.724	21.74	12.00	9.74	0.366	4.25	0.377
**Acute myocardial infarction**										
Aspirin within 24 h	81.82	87.88	−6.06	0.234	86.61	85.83	0.78	0.856	6.84	0.504
Aspirin at discharge	21.21	34.34	−13.13	0.039	35.43	20.47	14.96	0.008	28.09	0.530
β-blocker at discharge	11.11	23.23	−12.12	0.024	26.77	14.96	11.81	0.021	23.93	0.001
Smoking cessation advice	10.00	5.56	4.44	0.590	33.33	0.00	33.33	0.008	28.89	0.015
**Chronic asthma**										
Oxygenation index assessment	25.00	12.80	12.20	0.005	19.46	27.60	−8.14	0.044	−20.34	0.861
Influenza vaccine	0.00	0.00	0.00	0.000	0.45	0.00	0.45	0.317	0.45	0.317
Pneumonia vaccine	0.00	0.00	0.00	0.000	0.90	0.90	0.00	1.000	0.00	1.000
Smoking cessation advice	18.18	6.90	11.28	0.186	12.20	9.76	2.44	0.724	−8.84	0.250
**Stroke**										
Aspirin within 24 h	67.02	71.23	−4.21	0.277	72.79	73.81	−1.02	0.780	3.19	0.330
Aspirin at discharge	32.28	29.82	2.46	0.526	43.20	38.78	4.42	0.276	1.96	0.219
Statin at discharge	34.74	26.32	8.42	0.029	46.60	43.88	2.72	0.507	−5.70	0.050
Smoking cessation advice	29.09	0.00	29.09	0.000	22.22	2.04	20.18	0.002	−8.91	0.000

$${\mathrm{Y}}_{it}=\alpha +{\beta}_1{T}_1+{\beta}_2 RH+\delta {T}_1\ast RH+\omega X+\varepsilon$$

where Y_*it*_ is an indicator of process quality, 1 if treated and 0 otherwise. *T*_1_ =1 if the year is 2016 and 0 otherwise. RH = 1 if the patient’s hospital is a pilot hospital and 0 otherwise. *T*_1_ * *RH* is the DID variable and the coefficient δ represents the effect of the payment system reform. X represents control variable including patient age and whether the patient is a smoker. Results were estimated using a linear probability model, and coefficients can represent changes in service delivery rates.

#### Basic information

Table 1 shows the age distribution of patients with each disease. Pneumonia was predominated in patients aged < 19 years and over 61 years, accounting for 83.18% of all cases. Patients with acute myocardial infarction, stoke, and chronic asthma, were mainly over 41 years, accounting for more than 95% of all cases. Since the age of the patients was controlled at the PSM stage, it can be assumed that the age distribution of the control and intervention groups at each stage was consistent at the DID regression stage.

Table 2 shows the process quality of the control and intervention groups in different years. The overall difference in process quality between the intervention and control groups of patients with pneumonia was not significant at baseline and after the intervention. However, the intervention group had lower rates of antibiotic use before the intervention than the control group and the difference was smaller after the intervention. In contrast, smoking cessation advice rates were higher in the intervention group and further increased after the intervention.

For patients with acute myocardial infarction, the intervention group had lower rates of all service provisions than the control group at baseline, except for smoking cessation advice, but this was reversed after the intervention. The intervention group had a higher and increased rate of smoking cessation advice at baseline, while the control group became lower in 2016.

Vaccination rates for patients with chronic asthma were close to 0% both before and after the intervention. Rates of oxygenation index assessment and smoking cessation advice were higher in the intervention group at baseline, but improved in the control group and decreased in the intervention group for both indicators after the reform.

At baseline, the intervention group had a lower rate of aspirin treatment within 24 h for stroke patients (4.2%) and higher rates of other quality indicators. By 2016, levels of aspirin within 24 h were comparable in both groups, while the intervention group had higher rates of other service provisions.

#### Main results

Table 3 shows the main results of the DID regression. The intervention resulted in an 8.4% increase in the rate of sputum culture in patients with pneumonia(*p* < 0.001). The rates of antibiotics use and smoking cessation advice in patients with pneumonia increased (1.0, 3.8%), the rate of oxygenation index assessment and antibiotic use within 6 h in patients with pneumonia decreased (2.3, 1.4%), but none of these changes were statistically significant.

**Table 3:** Effect of GB combined with PFP on process quality using DID methods

QoC indicators	***β***_**1**_(SE)	***β***_**2**_(SE)	***δ***(SE)
**Pneumonia**			
Oxygenation index assessment	0.033(0.011) ***	−0.011(0.010)	−0.023(0.015)
Rate of sputum culture	−0.019(0.019)	−0.001(0.018)	0.084(0.027) ***
Antibiotic use	0.000(0.010)	−0.020(0.009) **	0.010(0.014)
Antibiotic use within 6 h	0.013(0.014)	−0.045(0.014) ***	− 0.014(0.020)
Influenza vaccine	0.001(0.003)	0.001(0.003)	−0.006(0.005)
Pneumonia vaccine	0.003(0.001) **	0.000(0.001)	−0.003(0.002)
Smoking cessation advice	−0.109(0.144)	0.055(0.150)	0.038(0.193)
**Acute myocardial infarction**			
Aspirin within 24 h	− 0.017(0.047)	− 0.061(0.050)	0.040(0.067)
Aspirin at discharge	−0.146(0.060) **	− 0.131(0.063) **	0.280(0.085) ***
β-blocker at discharge	−0.093(0.053) *	−0.121(0.055) **	0.237(0.075) ***
Smoking cessation advice	−0.079(0.112)	0.039(0.099)	0.305(0.143) **
**Chronic asthma**			
Oxygenation index assessment	0.149(0.042) ***	0.122(0.045) ***	−0.203(0.060) ***
Influenza vaccine	0.000(0.004)	0.000(0.004)	0.005(0.005)
Pneumonia vaccine	0.009(0.007)	0.000(0.008)	0.000(0.011)
Smoking cessation advice	0.038(0.080)	0.137(0.086)	−0.116(0.113)
**Stroke**			
Aspirin within 24 h	0.024(0.038)	− 0.057(0.040)	0.047(0.055)
Aspirin at discharge	0.091(0.040) **	0.014(0.042)	0.030(0.058)
Statin at discharge	0.177(0.040) ***	0.083(0.043) *	−0.056(0.058)
Smoking cessation advice	0.017(0.073)	0.265(0.077) ***	−0.066(0.102)

***p < 0.001, **p < 0.05, *p < 0.1.The control variables were entered into the DID regression model and the regression coefficients are not shown here as matching was already done in the first stage

*β*_1_ is the coefficient of the year, *β*_2_ is the coefficient of the intervention, and *δ* is the coefficient of the DID. *SE* standard error

The implementation of the GB system resulted in a 28% (*p* < 0.05) increase in aspirin use at discharge in acute myocardial infarction inpatients, a 23.7% (*p* < 0.05) increase in discharge with β-blocker, and a 30.5% (p < 0.05) increase in smoking cessation advice.

After implementation of the GB payment system, there was a large reduction in oxygenation index assessment in patients with chronic asthma (20.3%, *p* < 0.05), a 1.2% reduction in advising smoking cessation, but not statistically significant, and little change in influenza and pneumococcal vaccine use rates.

For stroke, there was an increase in the rate of aspirin within 24 h and aspirin at discharge (4.7 and 3.0%), and a decrease in statin at discharge and rate of smoking cessation advice (5.6 and 6.6%), and none of these changes were statistically significant.

The implementation of GB resulted in a decrease in oxygenation index assessment in patients with pneumonia (2.3%, *P* > 0.1) and a 20.3% decrease in oxygenation index assessment in patients with chronic asthma (*P* < 0.001).

The trend of change in aspirin treatment was the same in patients with acute myocardial infarction and stroke. Implementation of GB increased aspirin within 24 h by 40% in patients with acute myocardial infarction (*P* > 0.1) and by 4.7% in patients with stroke (P > 0.1). Rates of aspirin at discharge were 28% higher in patients with acute myocardial infarction (*P* < 0.001) and 3% higher in patients with stroke (*P* > 0.1).

For the rate of smoking cessation advice, different results were observed in this study. After implementation of GB, there was a 3.8% increase in patients with pneumonia (*P* > 0.1), a 30.5% increase in patients with acute myocardial attack (*P* < 0.01), a 11.6% decrease in patients with chronic asthma (*P* > 0.1) and a 6.6% decrease in patients with stroke (P > 0.1).

## Discussion

Previous study has found that the reform from FFS to GB has given providers a strong incentive to control costs, thus may bringing risks to the quality of care (QoC) [7, 34, 35]. However, in Guizhou, implementing global budget in county hospitals has improved the quality of care in hospitalized patients with acute myocardial infarction and pneumonia, which benefited from the supporting design of paying according to quality results, i.e., 30% of the budget was set aside as a bonus for the QoC assessment. Only when the QoC can be maintained or even improved, would the full amount of the budget be paid.

We found that quality improvements were mainly centered on patients with acute myocardial infarction. This may be due to a lack of knowledge of guidelines for the treatment of less life-threatening diseases among medical staff in county hospitals [36]. Better implementation of guidelines at the county level hospitals is an important safeguard for quality of care, and this may be a reminder that enhancing the training of healthcare workers’ skills should not be overlooked when undertaking payment reform.

There have been GB reforms elsewhere in China, but few accompanying arrangements have been made to ensure the quality of care. The same is true for other types of payment system reforms such as DRG. Payers have focused their attention almost entirely on cost control. The risks to health care quality are enormous and need to be adequately addressed. The practice of GB payment system in Guizhou province is worth learning from. In fact, many international reforms of the prepayment system also have similar institutional arrangements with quality control. For example, the UK’s Commissioning for Quality and Innovation for dementia care monitors service quality indicators and will only pay for a single service if it reaches 90%. In Germany, a large insurance company has a quality payment contract with a hospital in Karlsruhe that pays more for bypass surgery if in-hospital mortality and post-operative mediastinitis are below the German average [37]. Netherland insurers, on the other hand, use 30% of the total amount to negotiate quantity, quality and price [4].

In Germany, the quality of care has increased after the implementation of the prepayment reform. This is because the prepayment reform was accompanied by an increase in the quality of medical data, which made it easier and more reliable to use claim data for quality analysis [38]. The method of quality assessment of county hospitals in Guizhou province uses chart review, which is more costly. The local health insurance records few variables in the claim data, especially lacking accurate records of the patient’s arrival and discharge, what treatment was received during the hospital stay, what drugs were used, and the time when these treatments/drugs were received, so it is not possible to carry out a convenient process quality analysis using the established data. Once quality assessments are difficult and costly, payment based on quality assessment results can be problematic in terms of sustainability.

China’s health insurance sector should learn from this experience and strive to improve the quality of health care data to provide more protection for the payment system reform. When data availability and measurability are met, new QoC indicators can be designed for dynamic evaluation and be incorporated into the PFP evaluation indicator system.

## Conclusions

1. The ultimate goal of payment system reform is to control health care costs, which inevitably induces physicians to use fewer resources. Managers need to take reasonable measures when designing payment reform programs, and pay attention to the quality risks that may arise under the new incentive mechanisms. Design reasonable quality assessment programs, and link them to the final payment, so as to ensure that cost control will not bring about quality reduction.

2. Quality monitoring and control needs the corresponding information system as a support, but unfortunately, well-developed information system is not common at present. Each assessment requires additional data collection, and will increase a lot of costs, resulting in difficulty in implementing the assessment and inaccurate results. The reform should consider configuring or improving the information system for quality monitoring to improve the quality of health insurance data and ensure the sustainability of the system. Once the electronic medical record system is further developed, the services received by patients can be dynamically observed, allowing for more continuous and complete quality monitoring.

## Limitations

1. It would be more convincing to distinguish the effects of GB and PFP, but in this study the two interventions were present simultaneously. Therefore, we can only analyze the effect of GB combined with PFP on quality of care and compare it with other studies where only one intervention was implemented.

2. Due to the limited number of years of data, we could only use the PSM method to ensure that the characteristics of patients in the intervention and control groups were comparable, and tests, such as the test for homogeneous trend, were obstructed. As we are using data from the chart review, we also have no way to analysis medical costs.

## Supplementary Information

**Additional file 1:.**

**Additional file 2:.**

**Additional file 3:.**

**Additional file 4:.**

**Additional file 5:.**

## Data Availability

The datasets generated and/or analyzed during the current study are not publicly available due to legal restrictions and confidential nature of the data but are available from the corresponding author on reasonable request.

## Abbreviations

GB: global budget
PFP: Pay For performance
FFS: fee For services
QoC: QUALITY of care
SE: Standard error

## References

[1] Yip W, Hsiao W (2014). Harnessing the privatisation of China's fragmented health-care delivery. Lancet.

[2] Yip W, Powell-Jackson T, Chen W, Hu M, Fe E, Hu M, Jian W, Lu M, Han W, Hsiao WC (2014). Capitation combined with pay-for-performance improves antibiotic prescribing practices in rural China. Health Aff.

[3] Jian W, Yip W, Hsiao W (2014). All-wined policy designing cases: responsibility system of Yinchi County-level Hospital in Ningxia. Chinese Health Economics.

[4] Tan SS, Ineveld M, Redekop K, Hakkaart- van Roijen L: the Netherlands: the diagnose behandeling combinaties. Diagnosis-related groups in Europe: moving towards transparency, efficiency and quality in hospitals 2011:425–446.

[5] Ko YL, Wang JW, Hsu HM, Kao CH, Lin CY (2018). What happened to health service utilization, health care expenditures, and quality of care in patients with acute pancreatitis after implementation of global budgeting in Taiwan?. Medicine (Baltimore).

[6] Delanois RE, Wilkie WA, Mohamed NS, Remily EA, Pollak AN, Mont MA: Maryland's global budget revenue model: how do costs and readmission rates fare for patients undergoing Total knee Arthroplasty? J knee Surg 2020. 10.1055/s-0040-1709677 32369838

[7] Neumann A, Baum F, Seifert M, Schoffer O, Kliemt R, March S, Häckl D, Swart E, Pfennig A, Schmitt J (2021). Reduction of days in inpatient Care in Psychiatric Hospitals with flexible and integrated treatment for patient-centered care with a global budget - results with three-year follow-up from the evaluation study EVA64. Psychiatr Prax.

[8] Delanois RE, Gwam CU, Cherian JJ, Etcheson JI, George NE, Schneider KA, Mont MA (2018). Global budget revenue on a single Institution's costs and outcomes in patients undergoing Total hip Arthroplasty. J Arthroplast.

[9] Sharfstein JM, Stuart EA, Antos J (2018). Global budgets in Maryland: assessing results to date. Jama.

[10] He R, Miao Y, Ye T, Zhang Y, Tang W, Li Z, Zhang L (2017). The effects of global budget on cost control and readmission in rural China: a difference-in-difference analysis. J Med Econ.

[11] Lin CY, Ma T, Lin CC, Kao CH (2016). The impact of global budgeting on health service utilization, health care expenditures, and quality of care among patients with pneumonia in Taiwan. Eur J Clin Microbiol Infect Dis.

[12] Song Z, Fendrick AM, Safran DG, Landon B, Chernew ME (2013). Global budgets and technology-intensive medical services. Healthc (Amst).

[13] Song Z, Rose S, Safran DG, Landon BE, Day MP, Chernew ME (2014). Changes in health care spending and quality 4 years into global payment. N Engl J Med.

[14] Song Z, Colla CH (2016). Specialty-based global payment: a new phase in payment reform. Jama.

[15] Song Z, Ji Y, Safran DG, Chernew ME (2019). Health care spending, utilization, and quality 8 years into global payment. N Engl J Med.

[16] Kan K, Li SF, Tsai WD (2014). The impact of global budgeting on treatment intensity and outcomes. Int J Health Care Finance Econ.

[17] Yan J, Lin HH, Zhao D, Hu Y, Shao R (2019). China's new policy for healthcare cost-control based on global budget: a survey of 110 clinicians in hospitals. BMC Health Serv Res.

[18] Gaspar K, Portrait F, van der Hijden E, Koolman X (2020). Global budget versus cost ceiling: a natural experiment in hospital payment reform in the Netherlands. Eur J Health Econ.

[19] Jian W, Lu M, Liu G, Chan KY, Poon AN (2019). Beijing's diagnosis-related group payment reform pilot: impact on quality of acute myocardial infarction care. Soc Sci Med.

[20] Musgrove P (1993). Investing in health: the 1993 world development report of the World Bank. Bull Pan Am Health Organ.

[21] Peabody JW, Quimbo S, Florentino J, Shimkhada R, Javier X, Paculdo D, Jamison D, Solon O (2017). Comparative effectiveness of two disparate policies on child health: experimental evidence from the Philippines. Health Policy Plan.

[22] Bardach NS, Wang JJ, De Leon SF, Shih SC, Boscardin WJ, Goldman LE, Dudley RA (2013). Effect of pay-for-performance incentives on quality of care in small practices with electronic health records: a randomized trial. Jama.

[23] Navathe AS, Volpp KG, Caldarella KL, Bond A, Troxel AB, Zhu J, Matloubieh S, Lyon Z, Mishra A, Sacks L (2019). Effect of financial Bonus size, loss aversion, and increased social pressure on physician pay-for-performance: a randomized clinical trial and cohort study. JAMA Netw Open.

[24] Andersen CT, Ahmadzai H, Rasekh AW, Akala FA, Haque T, Johnson R, Loevinsohn B, Sayed GD, Chopra M (2021). Improving health service delivery in conflict-affected settings: lessons from a nationwide strategic purchasing mechanism in Afghanistan. J Glob Health.

[25] Rob U, Alam MM (2013). Performance-based incentive for improving quality of maternal health services in Bangladesh. Int Q Community Health Educ.

[26] Peabody JW, Shimkhada R, Quimbo S, Solon O, Javier X, McCulloch C (2014). The impact of performance incentives on child health outcomes: results from a cluster randomized controlled trial in the Philippines. Health Policy Plan.

[27] Menya D, Platt A, Manji I, Sang E, Wafula R, Ren J, Cheruiyot O, Armstrong J, Neelon B, O'Meara WP (2015). Using pay for performance incentives (P4P) to improve management of suspected malaria fevers in rural Kenya: a cluster randomized controlled trial. BMC Med.

[28] Binyaruka P, Robberstad B, Torsvik G, Borghi J (2018). Does payment for performance increase performance inequalities across health providers? A case study of Tanzania. Health Policy Plan.

[29] Xu H, Yang Y, Wang C, Yang J, Li W, Zhang X, Ye Y, Dong Q, Fu R, Sun H (2020). Association of Hospital-Level Differences in care with outcomes among patients with acute ST-segment elevation myocardial infarction in China. JAMA Netw Open.

[30] Zhou MG, Wang HD, Zhu J, Chen WQ, Wang LH, Liu SW, Li YC, Wang LJ, Liu YN, Yin P (2016). Cause-specific mortality for 240 causes in China during 1990-2013: a systematic subnational analysis for the global burden of disease study 2013. Lancet.

[31] Cao B, Huang Y, She D-Y, Cheng Q-J, Fan H, Tian X-L, Xu J-F, Zhang J, Chen Y, Shen N (2018). Diagnosis and treatment of community-acquired pneumonia in adults: 2016 clinical practice guidelines by the Chinese thoracic society. Chinese Medical Association Clinical Respiratory J.

[32] Boulet LP, Reddel HK, Bateman E, Pedersen S, FitzGerald JM, O'Byrne PM (2019). The global initiative for asthma (GINA): 25 years later. Eur Resp J.

[33] Chinese Society of N (2018). Chinese stroke S: Chinese guidelines for diagnosis and treatment of acute ischemic stroke 2018. Chin J Neurol.

[34] Petrou P (2016). Global budget for Cyprus' National Health System: the promised land or a no Man's land?. Value Health Reg Issues.

[35] Schwarz J, Galbusera L, Bechdolf A, Birker T, Deister A, Duve A, Heiser P, Hojes K, Indefrey S, Johne J (2020). Changes in German mental health care by implementing a global treatment budget-a mixed-method process evaluation study. Front Psychiatry.

[36] Wang Y, Li Z, Zhao X, Wang C, Wang X, Wang D, Liang L, Liu L, Wang C, Li H (2018). Effect of a multifaceted quality improvement intervention on hospital personnel adherence to performance measures in patients with acute ischemic stroke in China: a randomized clinical trial. JAMA.

[37] Busse R, Geissler A, Aaviksoo A, Cots F, Häkkinen U, Kobel C, Mateus C, Or Z, O'Reilly J, Serdén L (2013). Diagnosis related groups in Europe: moving towards transparency, efficiency, and quality in hospitals?. Bmj.

[38] Velasco Garrido M, Gerhardus A, Røttingen JA, Busse R (2010). Developing health technology assessment to address health care system needs. Health Policy.

