# Supplementary figures and images for: Impact of global budget combined with pay-for-performance on the quality of care in county hospitals: a difference-in-differences study design with a propaensity-score-matched control group using data from Guizhou province, China
Source: BMC Health Serv Res. 2021 Dec 2;21:1296. doi: 10.1186/s12913-021-07338-8 (PMC8641159; doi:10.1186/s12913-021-07338-8)

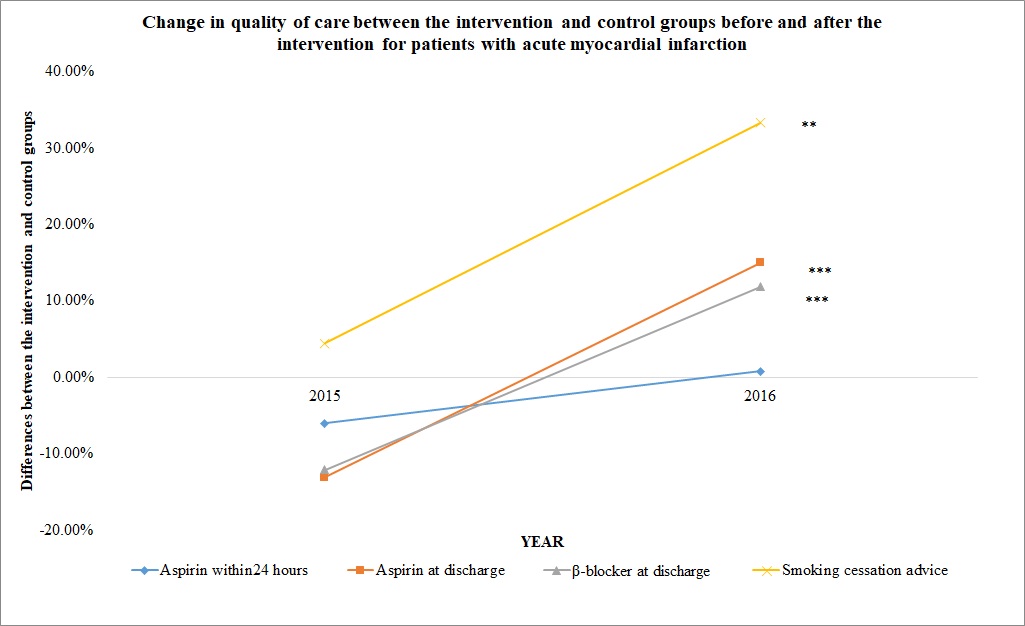

Supplement: Supplementary file 1 — Additional file 1:. [file 12913_2021_7338_MOESM1_ESM.jpg]

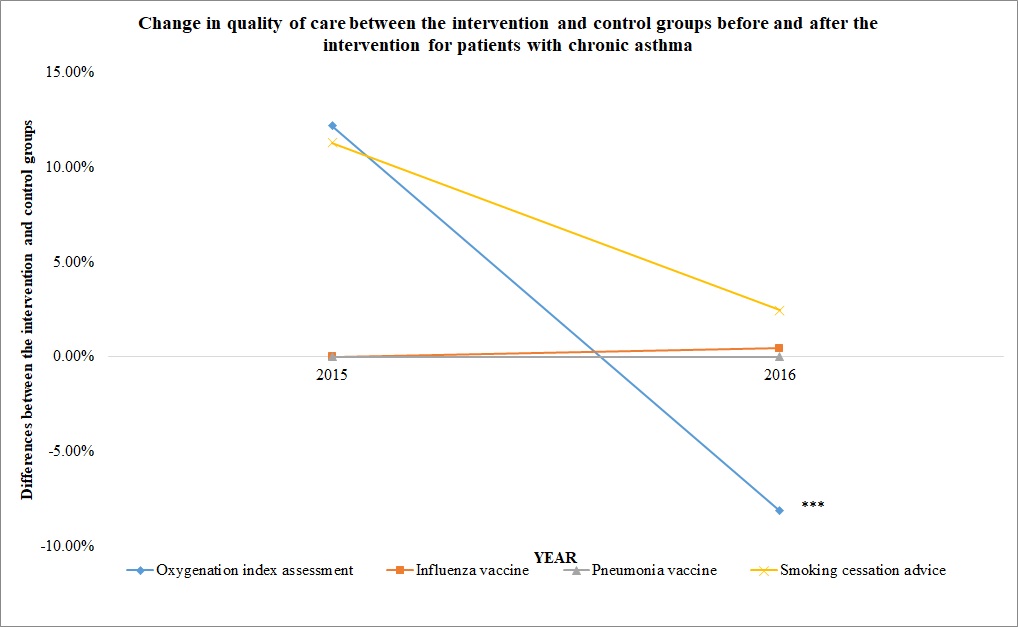

Supplement: Supplementary file 2 — Additional file 2:. [file 12913_2021_7338_MOESM2_ESM.jpg]

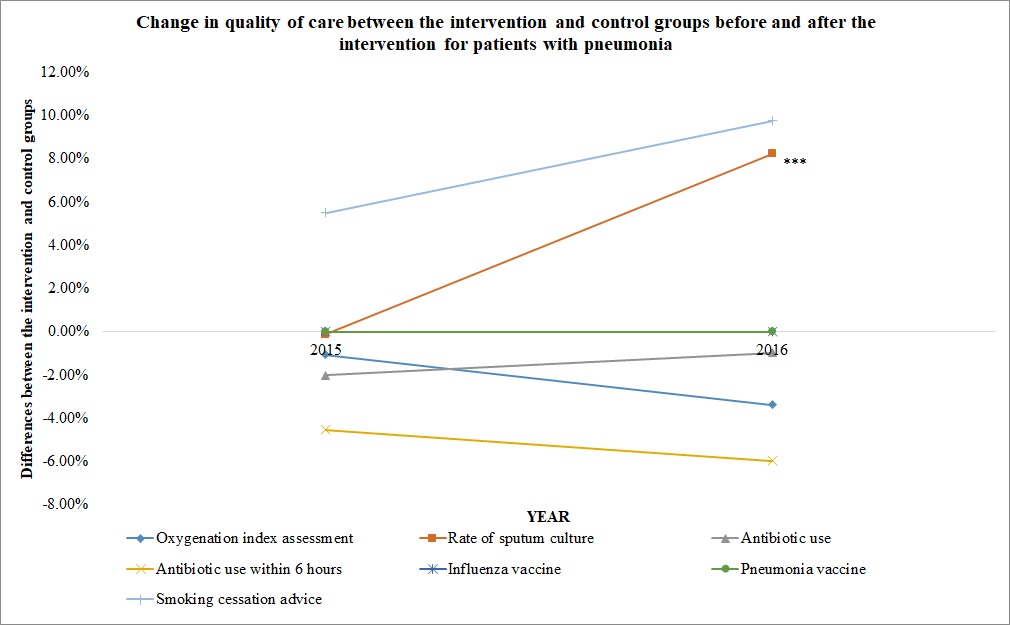

Supplement: Supplementary file 3 — Additional file 3:. [file 12913_2021_7338_MOESM3_ESM.jpg]

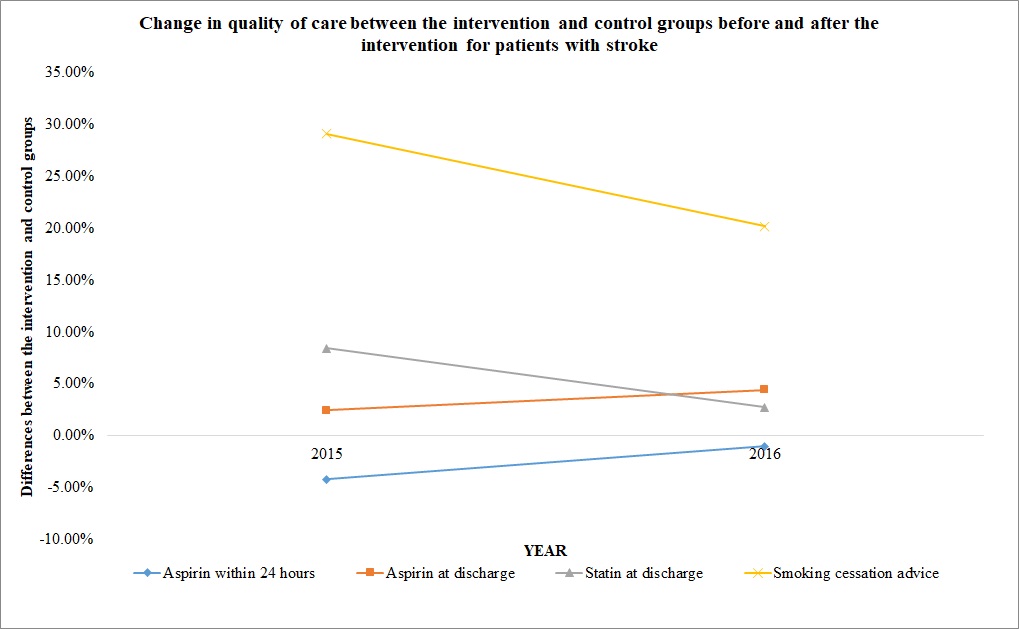

Supplement: Supplementary file 4 — Additional file 4:. [file 12913_2021_7338_MOESM4_ESM.jpg]
