# Supplementary material for: Impact of global budget combined with pay-for-performance on the quality of care in county hospitals: a difference-in-differences study design with a propaensity-score-matched control group using data from Guizhou province, China
Source: BMC Health Serv Res. 2021 Dec 2;21:1296. doi: 10.1186/s12913-021-07338-8 (PMC8641159; doi:10.1186/s12913-021-07338-8)
Supplement: Supplementary file 5 — Additional file 5:. [file 12913_2021_7338_MOESM5_ESM.docx]

| Table S1. Changes in patient characteristics before and after PSM matching | | | | | | | |
| --- | --- | --- | --- | --- | --- | --- | --- |
| case type | Variable | Unmatched  Matched | Mean | | t-test | | V(T)/  V© |
|  |  |  | Treated | Control | t | p>t |  |
| 2015  pneumonia | age group | 0 | 1.67 | 1.88 | -3.93 | 0.00 | 0.77* |
|  |  | 1 | 1.85 | 1.87 | -0.28 | 0.78 | 0.98 |
|  | smoke history | 0 | 0.02 | 0.02 | 0.83 | 0.41 | - |
|  |  | 1 | 0.01 | 0.02 | -1.10 | 0.27 | - |
| 2015  AMI | age group | 0 | 3.84 | 3.77 | 0.75 | 0.45 | 0.77 |
|  |  | 1 | 3.87 | 3.83 | 0.29 | 0.77 | 0.96 |
|  | smoke history | 0 | 0.29 | 0.18 | 1.91 | 0.06 | - |
|  |  | 1 | 0.18 | 0.19 | -0.19 | 0.85 | - |
| 2015  chronic asthma | age group | 0 | 4.01 | 3.92 | 1.64 | 0.10 | 0.88 |
|  |  | 1 | 3.92 | 3.92 | 0.00 | 1.00 | 1 |
|  | smoke history | 0 | 0.19 | 0.18 | 0.41 | 0.68 | - |
|  |  | 1 | 0.01 | 0.18 | -5.29 | 0.00 | - |
| 2015  stoke | age group | 0 | 3.85 | 3.84 | 0.31 | 0.75 | 0.9 |
|  |  | 1 | 3.26 | 3.84 | -10.86 | 0.00 | 0.68* |
|  | smoke history | 0 | 0.17 | 0.13 | 1.59 | 0.11 | - |
|  |  | 1 | 0.13 | 0.13 | 0.00 | 1.00 | - |
| 2016  pneumonia | age group | 0 | 1.73 | 1.92 | -3.24 | 0.00 | 0.81* |
|  |  | 1 | 1.95 | 1.92 | 0.34 | 0.74 | 1.05 |
|  | smoke history | 0 | 0.03 | 0.04 | -1.18 | 0.24 | - |
|  |  | 1 | 0.01 | 0.04 | -2.79 | 0.01 | - |
| 2016  AMI | age group | 0 | 3.82 | 3.82 | 0.02 | 0.99 | 0.8 |
|  |  | 1 | 3.57 | 3.89 | -4.26 | 0.00 | 0.63* |
|  | smoke history | 0 | 0.17 | 0.13 | 0.93 | 0.35 | - |
|  |  | 1 | 0.14 | 0.14 | 0.00 | 1.00 | - |
| 2016  chronic asthma | age group | 0 | 4.02 | 3.95 | 1.42 | 0.16 | 0.89 |
|  |  | 1 | 3.95 | 3.95 | 0.00 | 1.00 | 1 |
|  | smoke history | 0 | 0.18 | 0.19 | -0.12 | 0.91 | - |
|  |  | 1 | 0.36 | 0.18 | 4.36 | 0.00 | - |
| 2016  stoke | age group | 0 | 3.84 | 3.89 | -1.17 | 0.24 | 1.17* |
|  |  | 1 | 3.77 | 3.89 | -2.59 | 0.01 | 0.99 |
|  | smoke history | 0 | 0.15 | 0.17 | -1.04 | 0.30 | - |
|  |  | 1 | 0.35 | 0.17 | 5.06 | 0.00 | - |

| Table S2. Effect of GB combined with PFP on process quality using DID methods. ( probit ) | | | |
| --- | --- | --- | --- |
| Quality indicators | probit | | |
|  | $\boldsymbol{\beta}_{\boldsymbol{1}}$**(SE)** | $\boldsymbol{\beta}_{\boldsymbol{2}}$**(SE)** | $\boldsymbol{\delta}$**(SE)** |
| **Pneumonia** |  |  |  |
| Oxygenation index assessment | 0.348(0.226) | -0.129(0.344) | -0.250(0.417) |
| Rate of sputum culture | -0.093(0.0904) | -0.011(0.394) | 0.351(0.191)* |
| Antibiotic use | 0.007(0.164) | -0.261(0.336) | 0.109(0.179) |
| Antibiotic use within 6 hours | 0.137(0.221) | -0.318(0.310) | -0.143(0.252) |
| Influenza vaccine | 0.063(0.155) | 0.084(0.249) |  |
| Pneumonia vaccine |  |  |  |
| Smoking cessation advice | -0.439(0.477) | 0.163(0.606) | 0.220(0.582) |
| **Acute myocardial infarction** |  |  |  |
| Aspirin within 24 hours | -0.07(0.188) | -0.247(0.260) | 0.158(0.227) |
| Aspirin at discharge | -0.45(0.293) | -0.400(0.314) | 0.854(0.384)** |
| β-blocker at discharge | -0.357(0.273) | -0.496(0.256)* | 0.913(0.354)*** |
| Smoking cessation advice | -4.307(0.496)*** | 0.310(0.621) | 5.145(0.633)*** |
| **Chronic asthma** |  |  |  |
| Oxygenation index assessment | 0.552(0.425) | 0.469(0.363) | -0.737(0.462) |
| Influenza vaccine |  |  |  |
| Pneumonia vaccine |  |  | 0.000(0.374) |
| Smoking cessation advice | 0.232(0.375) | 0.694(0.533) | -0.563(0.507) |
| **Stroke** |  |  |  |
| Aspirin within 24 hours | 0.072(0.181) | -0.167(0.173) | 0.136(0.227) |
| Aspirin at discharge | 0.248(0.152) | 0.042(0.215) | 0.072(0.198) |
| Statin at discharge | 0.485(0.16)*** | 0.240(0.291) | -0.171(0.217) |
| Smoking cessation advice | 3.447(0.375)*** | 4.840(0.348)*** | -3.532(0.392)*** |
| ***p<0.001, **p<0.05, *p<0.1. The control variables were entered into the DID regression model and the regression coefficients are not shown here as matching was already done in the first stage.  $\beta_{1}$ is the coefficient of the year, $\beta_{2}$ is the coefficient of the intervention, and $\delta$ is the coefficient of the DID. SE: standard error. | | | |

| Table S3. Effect of GB combined with PFP on process quality using DID methods. ( logistic ) | | | |
| --- | --- | --- | --- |
| Quality indicators | logistic | | |
|  | $\boldsymbol{\beta}_{\boldsymbol{1}}$**(SE)** | $\boldsymbol{\beta}_{\boldsymbol{2}}$**(SE)** | $\boldsymbol{\delta}$**(SE)** |
| **Pneumonia** |  |  |  |
| Oxygenation index assessment | 0.682(0.533) | -0.366(0.811) | -0.351(1.012) |
| Rate of sputum culture | -0.169(0.166) | -0.011(0.737) | 0.636(0.348)* |
| Antibiotic use | -0.000(0.366) | -0.567(0.750) | 0.251(0.396) |
| Antibiotic use within 6 hours | 0.279(0.475) | -0.639(0.636) | -0.298(0.527) |
| Influenza vaccine | 0.184(0.457) | 0.289(0.779) |  |
| Pneumonia vaccine | |  |  |
| Smoking cessation advice | -0.776(0.848) | 0.286(1.040) | 0.399(1.053) |
| **Acute myocardial infarction** | | |  |
| Aspirin within 24 hours | -0.158(0.349) | -0.492(0.482) | 0.314(0.418) |
| Aspirin at discharge | -0.753(0.502) | -0.669(0.544) | 1.425(0.659)** |
| β-blocker at discharge | -0.624(0.482) | -0.898(0.469)* | 1.616(0.626)*** |
| Smoking cessation advice | | |  |
| **Chronic asthma** | |  |  |
| Oxygenation index assessment | 0.963(0.755) | 0.822(0.663) | -1.280(0.817) |
| Influenza vaccine | |  |  |
| Pneumonia vaccine | |  | 0.000(0.972) |
| Smoking cessation advice | 0.462(0.755) | 1.318(0.970) | -1.095(0.975) |
| **Stroke** |  |  |  |
| Aspirin within 24 hours | 0.120(0.305) | -0.276(0.287) | 0.224(0.381) |
| Aspirin at discharge | 0.406(0.246)* | 0.070(0.355) | 0.113(0.322) |
| Statin at discharge | 0.791(0.254)*** | 0.393(0.482) | -0.283(0.348) |
| Smoking cessation advice | | |  |
| ***p<0.001, **p<0.05, *p<0.1. The control variables were entered into the DID regression model and the regression coefficients are not shown here as matching was already done in the first stage.  $\beta_{1}$ is the coefficient of the year, $\beta_{2}$ is the coefficient of the intervention, and $\delta$ is the coefficient of the DID. SE: standard error. | | | |
